# Specific Activation of A_3_, A_2A_ and A_1_ Adenosine Receptors in CD73-Knockout Mice Affects B16F10 Melanoma Growth, Neovascularization, Angiogenesis and Macrophage Infiltration

**DOI:** 10.1371/journal.pone.0151420

**Published:** 2016-03-10

**Authors:** Patrycja Koszałka, Monika Gołuńska, Aleksandra Urban, Grzegorz Stasiłojć, Marcin Stanisławowski, Marceli Majewski, Andrzej C. Składanowski, Jacek Bigda

**Affiliations:** 1 Laboratory of Cell Biology, Department of Medical Biotechnology, Intercollegiate Faculty of Biotechnology UG-MUG, Medical University of Gdańsk, Poland; 2 Department of Histology, Faculty of Medicine, Medical University of Gdańsk, Poland; 3 Laboratory of Molecular Enzymology, Department of Medical Biotechnology, Intercollegiate Faculty of Biotechnology UG-MUG, Medical University of Gdańsk, Poland; University of Bari Medical School, ITALY

## Abstract

CD73 (ecto-5'-nucleotidase), a cell surface enzyme hydrolyzing AMP to adenosine, was lately demonstrated to play a direct role in tumor progression including regulation of tumor vascularization. It was also shown to stimulate tumor macrophage infiltration. Interstitial adenosine, accumulating in solid tumors due to CD73 enzymatic activity, is recognized as a main mediator regulating the production of pro- and anti-angiogenic factors, but the engagement of specific adenosine receptors in tumor progression *in vivo* is still poorly researched. We have analyzed the role of high affinity adenosine receptors A_1_, A_2A_, and A_3_ in B16F10 melanoma progression using specific agonists (CCPA, CGS-21680 and IB-MECA, respectively). We limited endogenous extracellular adenosine background using CD73 knockout mice treated with CD73 chemical inhibitor, AOPCP (adenosine α,β-methylene 5’-diphosphate). Activation of any adenosine receptor significantly inhibited B16F10 melanoma growth but only at its early stage. At 14th day of growth, the decrease in tumor neovascularization and MAPK pathway activation induced by CD73 depletion was reversed by all agonists. Activation of A_1_AR primarily increased angiogenic activation measured by expression of VEGF-R2 on tumor blood vessels. However, mainly A_3_AR activation increased both the microvessel density and expression of pro-angiogenic factors. All agonists induced significant increase in macrophage tumor infiltration, with IB-MECA being most effective. This effect was accompanied by substantial changes in cytokines regulating macrophage polarization between pro-inflammatory and pro-angiogenic phenotype. Our results demonstrate an evidence that each of the analyzed receptors has a specific role in the stimulation of tumor angiogenesis and confirm significantly more multifaceted role of adenosine in its regulation than was already observed. They also reveal previously unexplored consequences to extracellular adenosine signaling depletion in recently proposed anti-CD73 cancer therapy.

## Introduction

Ecto-5’-nucleotidase (CD73, eN), a cell adhesion molecule and an enzyme catalyzing the conversion of 5'-AMP to bioactive extracellular adenosine is found to be upregulated in various types of cancer, including melanomas [1, 2]. CD73 knockout or inhibition/blockade of its activity was demonstrated to restrain the growth of subcutaneous tumors and metastasis formation [3–5]. Its role in tumor progression was reported as multifaceted one, recently including its influence on intratumoral microvessel density [1, 4, 6]. In solid tumors with no adequate blood supply adenosine accumulates and acts as a hypoxia-counteracting mediator initiating a range of tissue responses including regulation of angiogenesis process [7]. Dysregulation of angiogenesis is a hallmark of cancer and the process of angiogenic switch is crucial for tumor growth and invasiveness [8]. However, the role of adenosine in this process is still poorly researched in tumors *in vivo*, especially in view of the recently discussed anti-CD73 cancer therapy.

Most effects of adenosine are mediated through G-protein-coupled adenosine receptors, denoted A_1_, A_2A_, A_2B_, and A_3_ and classed as P1 purinergic receptors. Each of these receptors functions via a different internal effector mechanism, with activation of A_1_AR and A_3_AR resulting in decreased intracellular cAMP levels and activation of A_2A_AR and A_2B_AR in cAMP accumulation [9, 10]. At physiological conditions adenosine can activate high affinity A_1_, A_2A_, and A_3_ receptors (EC_50_ in the 0.2–0.7 μM range) but higher pathological concentrations of adenosine are needed to activate the low affinity A_2B_ receptors (EC_50_ ab. 24 μM) [9]. The main role of adenosine in angiogenesis was indicated to be its ability to regulate the production of pro- and anti-angiogenic factors, mediated predominantly through A_2A_ and A_2B_ receptors. Some reports also point out direct mitogenic effects of adenosine on vascular cells *in vitro* [7, 11, 12]. It was demonstrated in Lewis lung carcinoma *in vivo* that A_2B_AR-knockout decreased the density of intratumoral blood vessels and expression of vascular endothelial growth factor (VEGF) by host cells [13]. A_3_AR was shown to promote angiogenesis through a paracrine mechanism involving the differential expression and secretion of angiogenic factors from mast cells [14]. A_1_AR role in angiogenesis was indicated to be mainly indirect through non-vascular cells but its direct action on endothelial cells was not excluded [7, 15]. However, there is only one report of its role in angiogenesis *in vivo*, in naturally immunodeficient chicken chorioallantoic membrane (CAM) model [15] and its role in tumor angiogenesis was not studied in *in vivo* model.

Aiming to analyze the role of A_3_, A_2A_ and A_1_ adenosine receptors in the tumor growth and vascularization *in vivo*, we used CD73-knockout mice [16] treated with AOPCP to deplete melanoma endogenous adenosine background. This allowed us to stimulate single adenosine receptor with a selective agonist and to analyze its specific effect on analyzed processes.

Earlier we reported that in CD73-knockout mice inhibition of tumor CD73 with AOPCP (adenosine 5’-*alpha*,*beta*-methylene diphosphate) decreased B16F10 melanoma growth [4, 17]. Change in tumor growth correlated with significantly decreased microvessel density modified expression of pro-angiogenic factors in tumor microenvironment. However, it was also accompanied by significant decrease in angiogenic activation of endothelial cells in tumor blood vessels, a sharp decrease in tumor macrophage infiltration and changes in expression profile of factors influencing macrophage polarization [4].

Here we demonstrate an evidence that each of the analyzed receptors has a specific role in the stimulation of tumor angiogenesis. The changes in angiogenic activation of blood vessels observed in this model were shown to be mediated mainly through A_1_AR. However, it was A_3_AR that displayed the most powerful effect on development of blood vessels. All receptors, with the most significant role of A_3_, had an effect on both macrophage infiltration of melanoma and on cytokine expression profile in tumor microenvironment. This is also the first study to show a role of A_1_, A_2A_ and A_3_ adenosine receptors in the process of tumor vascularization *in vivo*, without the background of endogenous extracellular adenosine signaling.

## Materials and methods

### Mice

Wild-type (WT) and CD73-deficient (CD73^-/-^) C57BL/6 mice [16] were bred and maintained at the Tri-city Central Animal Laboratory of the Medical University of Gdansk (Poland) under SPF (specific pathogen free) conditions. Animal studies were performed on male 4- to 8-week-old mice. This study was carried out in strict accordance with the EU and national guidelines for the Care and Use of Laboratory Animals (Journal of Laws No. 33, pos. 289). The protocol was approved by the 3^rd^ Local Independent Ethics Committee in Gdańsk (Permit Number: 41/2008 and 32/2011). All efforts were made to minimize suffering. Both animal handling and procedures were supervised by experienced veterinarian observing animals for the development of adverse clinical signs, including labored breathing, weight loss, abdominal swelling, self-mutilation and paralysis. Animals were euthanized with isoflurane at a humane endpoint, at an early stage of subcutaneous tumor growth, before the onset of adverse clinical signs. No animals had to be sacrificed before the experimental endpoint due to the adverse clinical signs.

### Cell lines

The B16F10 murine melanoma cell line was kindly provided by Prof. J. Konopa (Gdańsk University of Technology, Poland) and acquired from ATCC (LGC Standards, Lomianki, Poland). Cells were cultured in DMEM (Sigma-Aldrich, St Louis, MO, USA) supplemented with 10% FBS (CytoGen, Lodz, Poland) in a humidified atmosphere containing 5% CO_2_ at 37°C.

### Reagents

Monoclonal anti-ERK1/2 and anti-pERK1/2 (Thr202/Tyr204) antibodies were purchased from Cell Signaling Technology (Danvers, MA, USA); anti-F4/80 from Novus Biologicals (Littleton, CO, USA); anti-CD31, anti-VEGF-R2, anti-Bad from BD Pharmingen (San Jose, CA, USA); anti-β-tubulin from Santa Cruz Biotechnology (Santa Cruz, CA, USA); recombinant Protein G-HRP conjugate from Thermo Scientific (Waltham, MA, USA). AOPCP (adenosine 5’-*alpha*,*beta*-methylene diphosphate), A_3_AR agonist IB-MECA (N^6^-(3-Iodobenzyl)adenosine-5′-N-methyluronamide, 1-Deoxy-1-[6-[((3-Iodophenyl)methyl)amino]-9H-purin-9-yl]-N-methyl-β-D-ribofuranuronamide), A_2A_AR agonist CGS-21680 (2-*p*-(2-Carboxyethyl)phenethylamino-5′-N-ethylcarboxamidoadenosine hydrochloride hydrate), A_1_AR agonist CCPA (2-Chloro-N^6^-cyclopentyladenosine) and cell culture reagents were purchased from Sigma-Aldrich (St Louis, MO, USA).

### In vivo treatments

To analyze tumor growth, B16F10 cells (2.5x10^6^) were injected subcutaneously into the left flank of syngenic C57BL/6 wild-type or CD73-deficient mice. Experimental groups included wild-type mice, DMSO-treated wild-type mice, AOPCP-treated CD73^-/-^ mice and AOPCP-treated CD73^-/-^ mice injected with one AR agonist (IB-MECA, CGS-21680 or CCPA). Tumor size was monitored with calipers each day for 14 days. Then, mice were anesthetized with isoflurane and euthanized. Tumors were excised and snap-frozen in liquid nitrogen for subsequent analyses. When indicated, mice were injected intraperitoneally with: 1. AOPCP at 20 mg/kg on days 5, 7, 9 and 12 2. AR agonist at 0.1 mg/kg every day or 3. DMSO at 0.02 ml/kg in saline (agonist vehicle) every day after s.c. injection of tumor cells. Stock solutions of agonists (5 mg/ml) were made in 100% DMSO and appropriately diluted in saline for injections.

### Immunohistochemical analysis

Analysis was performed as described previously [4]. Briefly, tissue sections of snap-frozen tumors were incubated with primary antibodies overnight at 4°C (final dilution 1:50 for CD31, 1:20 for VEGF-R2 and 1:400 for F4/80). As a negative control, the primary antibody was replaced with nonimmune serum in a similar dilution. Sections were then incubated with the appropriate secondary antibodies followed by visualization of immunoreactive cells with ImmPACT NovaRED Peroxidase Substrate Kit (Vector Laboratories, Burlingame, CA, USA). Slides were counterstained with hematoxylin according to standard protocols. Each slide was photographed randomly at 40x total magnification under an Eclipse TE300 microscope (Nikon, Tokyo, Japan) with image analysis software analySIS FIVE (Olympus Soft Imaging Solutions, Tokyo, Japan) to obtain 4–6 fields (3 mm^2^ each) per slide. For assessment of intratumoral microvascular density CD31-stained capillaries as well as CD31 positive endothelial cells or cell clusters were counted from all captured fields at high resolution using ImageJ software (NIH, Bethesda, MD, USA) independently by three scientists with experience in histological data analysis. The same assessment was done on consecutive cryosections for VEGF-R2 stained microvessels. Tumor macrophage infiltration was analyzed with ImageJ software on photographs from anti-F4/80 stained slides for a percentage of the stained areas using the ‘analyze particles’ tool. Images were transformed to RGB stacks and analyzed on blue channel with the threshold set manually for every image (values varied between 75 and 115). For each experimental group, three tumors were analyzed and the results were averaged.

### Western blot analysis

Tumor fragments left after preparing cryosections for IHC staining were precisely weighted, placed on ice and homogenized in 300 μl of RIPA buffer containing Complete Protease Inhibitor Cocktail (Roche Applied Sciences, Indianapolis, IN, USA) per 0.1 g of tissue. Homogenate was transferred to fresh tubes, incubated on ice for 30 min and centrifuged for 10 min at 17000×g. Part of obtained supernatant was snap-frozen for other analyses, and the rest was prepared for SDS-PAGE as described previously [17]. The method was modified by the use of recombinant Protein G-HRP conjugate in exchange for secondary antibody to eliminate endogenous antibody background.

### Antibody arrays

Tumor cell lysates at a concentration of 1 mg/ml were analyzed by RayBiotech (Norcross, GA, USA) with RayBio Mouse Cytokine Antibody Arrays 3 and 4 (G-series) for 62 and 34 cytokines spotted on a glass slide. The sensitivity of the antibodies present in the arrays to bind detected antigens was in the range 1–1,000 pg/ml. To determine the cytokine concentrations, the densities of individual spots were measured in relation to the signal positive control, spots with standardized amounts of biotinylated IgG. After subtracting the background, the data presented as optical density units were normalized according to the positive control densities. The fold changes between groups were calculated from the means for each protein separately, assuming that the average expression of such protein in all groups is equal to 1. For each group, analyzed samples were pooled from three tumors from three separate experiments.

### Statistical analysis

For antibody microarray the mean values from duplicates obtained from a single experiment were used, and the statistical differences between experimental groups were measured by the Student’s t-test. CV was used as a measure of relative variability. For other experiments, mean values were obtained from at least three separate experiments and reported as the mean (±SD). For statistical analysis, the Mann-Whitney test for two unpaired groups of a non-Gaussian population was used. *P* values <0.05 were considered significant.

## Results

### Specific activation of A_3_, A_2A_ or A_1_ adenosine receptors is important for subcutaneous B16F10 melanoma growth

**Fig 1:**
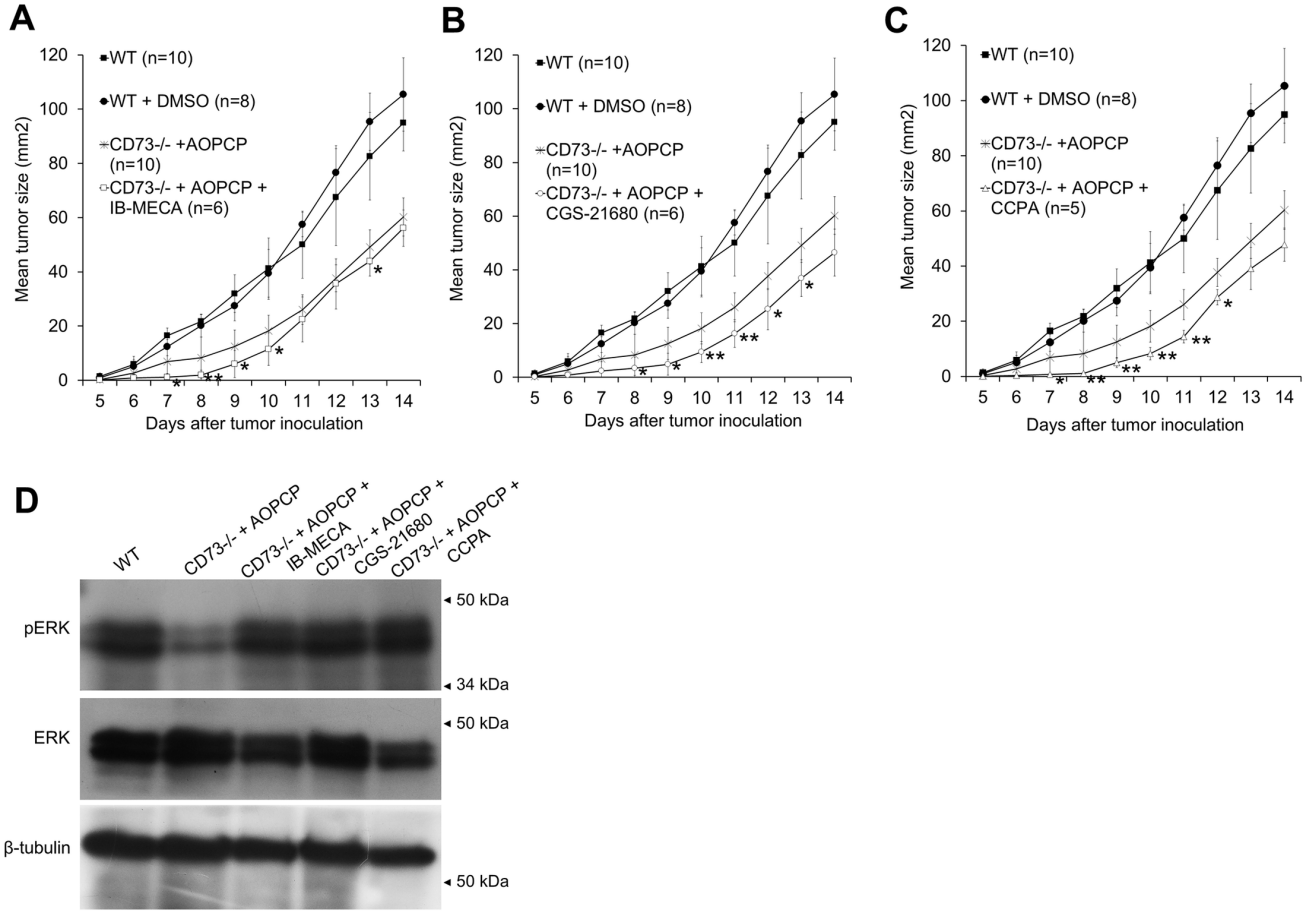
Stimulation of A_3_, A_2A_ or A_1_ adenosine receptors initially reduces subcutaneous B16F10 tumor growth but later activates MAP-kinase pathway. (A, B, C) Growth rate of B16F10 melanoma for 14 days post s.c. injection into WT or CD73^-/-^ mice. CD73^-/-^ mice were treated with AOPCP and, where indicated, with specific adenosine receptor agonist, IB-MECA (agonist for A_3_AR), CGS-2168 (A_2A_AR) or CCPA (A_1_AR). For separate control of agonist solvent, WT mice were treated with DMSO. Only statistically significant changes induced by agonist treatment compared to the CD73^-/-^ + AOPCP background are marked on the graph, *P<0.05, **P<0.01. (D) Expression of ERK1/2 and pERK1/2 on the 14^th^ day of tumor growth assessed by Western blot in tumor lysates. -tubulin shown as loading control.

To analyze the role of the individual adenosine receptors in B16F10 melanoma growth, we compared subcutaneous tumor growth rate in wild-type (WT), AOPCP-treated CD73 knockout mice (CD73^-/-^ + AOPCP) and AOPCP-treated CD73^-/-^ mice injected i.p. with specific A_1_, A_2A_, and A_3_ adenosine receptor agonists (CCPA, CGS-21680 or IB-MECA, respectively). DMSO-treated WT mice were used as a control for the possible effects of an agonist solvent. Tumor growth was monitored for 14 days as shown in Fig 1A–1C.

On the 14^th^ day there was no significant difference in size between growth-delayed tumors (P<0.01 from 7^th^ day) from AOPCP-treated CD73^-/-^ mice and tumors from such mice treated with adenosine receptor agonists. However, all agonists additionally inhibited tumor growth rate at an early stage (days 8–10) (P<0.01 for CCPA, P<0.01–0.05 for CGS-21680 and P<0.05 for IB-MECA) with the effect of CGS-21680 (Fig 1B) sustained until 13^th^ day from injection. DMSO was maintained then at the level corresponding to LD50/310 ratio [18] so its significant influence on tumor growth seems unlikely, especially that its influence on WT tumor growth was negligible.

The proliferative potential of tumor cells was evaluated on the 14^th^ day of tumor growth as a change in the levels of phosphorylated ERKs (pERK1/2), a downstream component of MAPK pathway (Fig 1D). At this stage of tumor growth, an activation of each analyzed adenosine receptor reversed the decrease of active ERKs induced when extracellular adenosine signaling was depleted. pERK1/2 were upregulated by agonists to the level similar as in WT tumors.

The above indicates that activation of A_3_, A_2A_ or A_1_ adenosine receptors is able to inhibit tumor growth only at its early stage, but at the more advanced stage proliferative activity of agonist-stimulated tumors is at a comparable level to wild-type tumors with intact CD73 activity.

### Specific activation of A_3_ and A_1_ adenosine receptors stimulates tumor neovascularization with mainly A_1_AR involved in angiogenic activation of existing blood vessels

**Fig 2:**
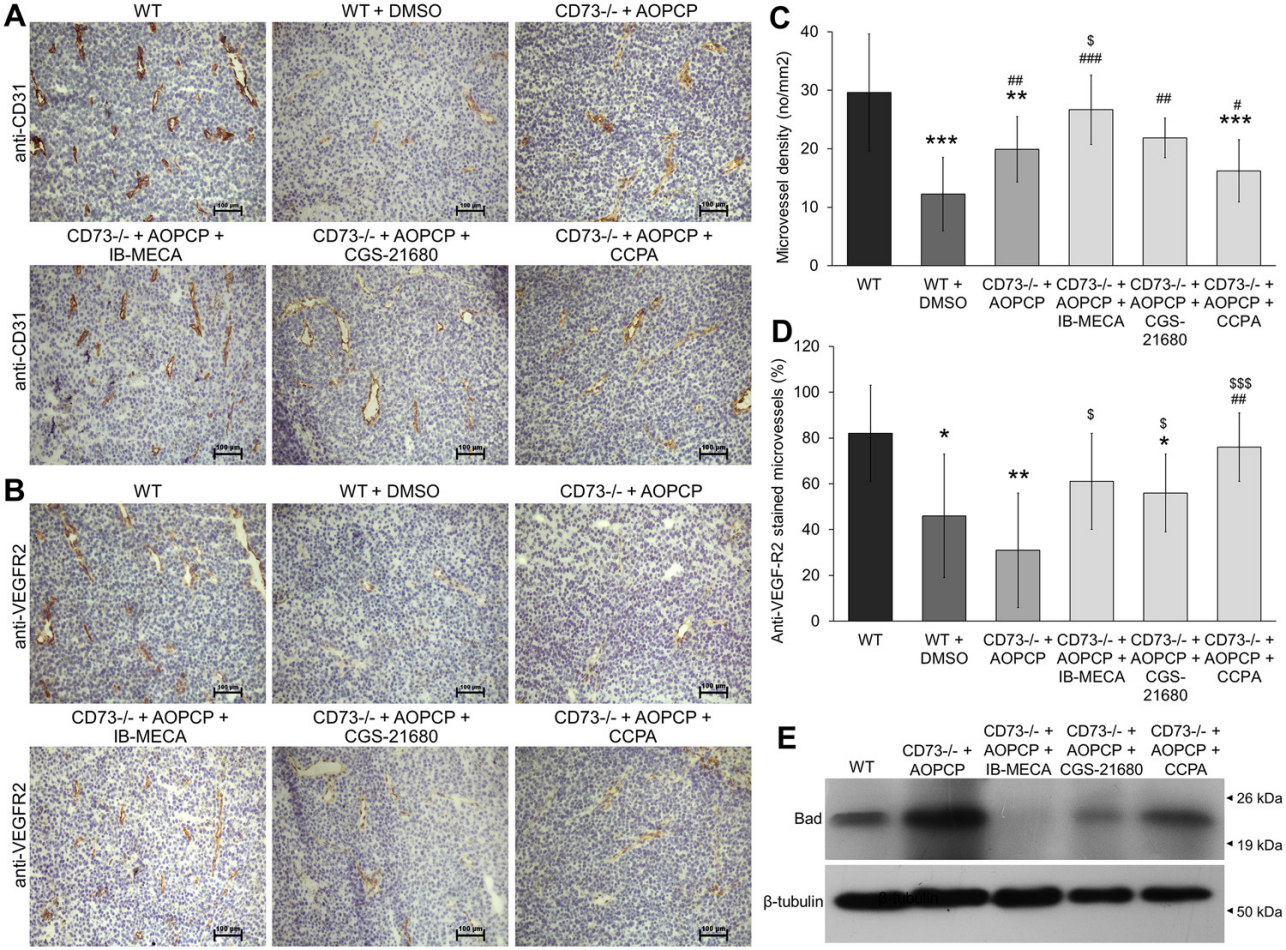
Stimulation of A_3_, A_2A_ or A_1_ adenosine receptors modulates density of intratumoral blood vessels and their angiogenic activation. (A, B) ICH staining of a pan-endothelial marker (CD31) and angiogenic activation marker (VEGF-R2) shown on consecutive slides. Tumors were analyzed on the 14^th^ day after s.c. B16F10 injection into WT or CD73^-/-^ mice. CD73 activity on tumor cells in CD73^-/-^ mice was inhibited by i.p. injections of AOPCP and, where indicated, adenosine receptors were stimulated with specific agonists, IB-MECA (agonist for A_3_AR), CGS-2168 (A_2A_AR) or CCPA (A_1_AR). Scale bar represents 100 μm. Slides were photographed at 100x total magnification under an Eclypse E800 microscope, DS-Fi1c camera and NIS-ELEMENTS AR, version 4.30 image analysis software (Nikon, Tokyo, Japan). (C) Microvessel density quantified as CD31-positive microvessels per mm^2^. (D) Percentage of CD31-positive microvessels expressing VEGF-R2 on a consecutive slides. 4–6 random fields were counted (magnification 40x, 3 mm^2^/field) per a slide from 3 tumors per a group. *P<0.05, **P<0.01, ***P<0.001 vs. WT, ^#^P<0.05, ^##^P<0.01, ^##^P<0.001 vs. WT + DMSO, ^$^P<0.05, ^$ $ $^P<0.001 vs. CD73^-/-^ + AOPCP. (E) Expression of protein Bad on the 14^th^ day of tumor growth assessed by Western blot in tumor lysates. -tubulin shown as loading control.

The role of signaling through adenosine receptors A_3_, A_2A_ and A_1_ on tumor angiogenesis in melanoma tumors was immunohistochemically assessed on the 14^th^ day after s.c. injection of B16F10 cells. WT, DMSO-treated WT, AOPCP-treated CD73^-/-^ mice and CD73^-/-^ mice treated with both AOPCP and one of the AR agonists (IB-MECA, CGS-21680 or CCPA) were analyzed. Both intratumoral microvessel density (iMVD) analyzed using anti-CD31 staining (Fig 2A and 2C), and angiogenic activation analyzed as a percentage of CD31-positive blood vessels expressing main pro-angiogenic receptor VEGF-R2 [19] (Fig 2B and 2D) were quantified.

In AOPCP-treated CD73^-/-^ mice, the inhibition of CD73 have led to the significant decrease in mean iMVD (20 microvessels /mm^2^, P<0.001) compared to the WT control (ab. 30 microvessels/mm^2^) as was reported earlier [4]. The decrease in mean iMVD was even stronger in DMSO-treated WT control, declining to 12 microvessels/mm^2^ (P<0.01 compared to WT). All agonists have significantly increased microvessel density. The strongest effect had an activation of A_3_AR as it augmented iMVD up to the level similar to that of WT tumors (27 microvessels/mm^2^, P<0.05 compared to CD73-depleted control, P<0.001 to the solvent control). Activation of A_2A_AR and A_1_AR only partially reversed the changes in microvessel density, with a significant increase only when compared to the solvent control (22 microvessels/mm^2^, P<0.01 and 16 microvessels/mm^2^, P<0.05, respectively). iMVD score of CCPA-treated tumors was still well below WT (P<0.001). The increase in microvascular density scores correlated with the decrease in expression of protein Bad (Fig 2E), a marker of metabolic changes due to the hypoxia [20, 21].

When angiogenic activation was analyzed, the specific stimulation of A_1_AR gave the strongest increase in percentage of VEGF-R2 stained blood vessels (76% of the overall number of microvessels) up to the level of WT tumors (82%). The change was statistically significant when compared to the strongly decreased VEGF-R2 expression on blood vessels in both background controls, DMSO-treated WT (46%, P<0.01) or AOPCP-treated CD73^-/-^ tumors (31%, P<0.001). Signaling through A_3_AR and A_2A_AR only partially increased this percentage (61% and 56%, respectively), reaching significance only when compared to the extracellular adenosine depleted tumors (P<0.05). CGS-21680 treated tumors showed an angiogenic activation of blood vessels still below WT level (P<0.05). This indicates a putative role of adenosine signaling in regulation of VEGF-R2 expression on blood vessels mainly through activation of A_1_AR. Known anti-angiogenic effect of agonist solvent, DMSO [22] did not mask specific effects of agonists in our experimental setup.

### Specific activation of A_3_, A_2A_ or A_1_ adenosine receptors modifies an angiogenesis-related protein expression profile in B16F10 melanoma

**Fig 3:**
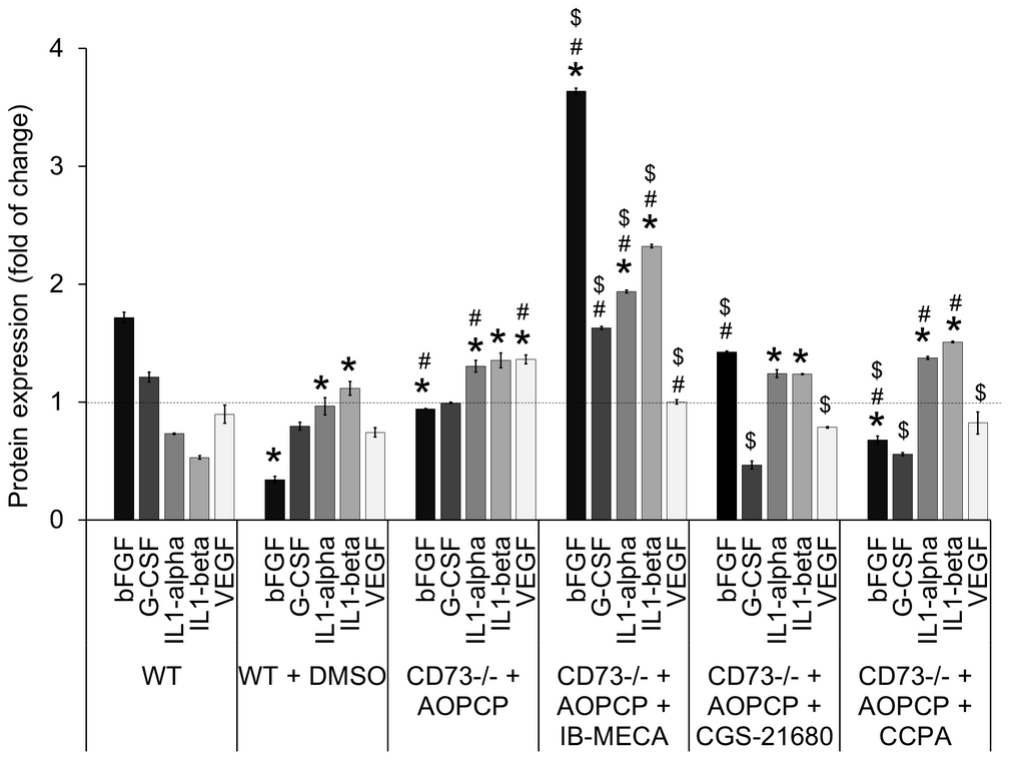
A_3_, A_2A_ or A_1_ adenosine receptors regulate expression of the main pro-angiogenic factors in B16F10 melanoma. Protein expression was analyzed in lysates from tumors on the 14^th^ day of growth in WT or CD73^-/-^ mice. CD73^-/-^ mice were treated with AOPCP and, where indicated, with specific adenosine receptor agonist, IB-MECA (agonist for A_3_AR), CGS-2168 (A_2A_AR) or CCPA (A_1_AR). For separate control of agonist solvent, WT mice were treated with DMSO. Semi-quantitative antibody microarrays were used to obtain protein expression profile presented as the fold change between groups calculated from the mean values for each protein separately, assuming that the average expression of the protein in all three control groups is equal to 1. *P<0.05 vs. WT, ^#^P<0.05 vs. WT + DMSO, ^$^P<0.05 vs. CD73^-/-^ + AOPCP (other statistical differences are not shown).

Semi-quantitative Mouse Cytokine Antibody Arrays (RayBiotech, Norcross, USA) were used to assess changes induced in tumor proteomes by specific stimulation of adenosine receptors in CD73-depleted mice as compared to WT, DMSO-treated WT and AOPCP-treated CD73^-/-^ mice. Ninety-six angiogenesis-related cytokines, chemokines, growth factors and receptors were detected in the range of 1–1,000 pg/ml in lysates of tumors isolated on the 14^th^ day after s.c. injection. Eighty-one proteins with significant expression differences between groups (P<0.05, Student’s t-test) were selected, and the fold changes in expression with 1 equal to the average expression of each protein in all three control groups were calculated. Fig 3 shows the changes in expression of five main pro-angiogenic factors [8, 23]: basic fibroblast growth factor (bFGF), granulocyte colony-stimulating factor (G-CSF), interleukins 1-alpha and 1-beta (IL1-alpha, IL1-beta) and vascular endothelial growth factor (VEGF; isoform 164).

In both background controls, DMSO-treated WT mice and AOPCP-treated CD73^-/-^ mice, the profile of main pro-angiogenic factors was different compared to WT control. Both background controls had decreased bFGF expression. IL1-alpha and IL1-beta were both upregulated, but significantly stronger in AOPCP-treated CD73^-/-^ tumors. In CD73-depleted tumors there was also significant increase in hypoxia-regulated VEGF, not observed in DMSO-treated WT tumors.

Specific activation of adenosine receptors in melanoma growing in CD73-depleted mice induced significant changes in expression of pro-angiogenic factors. Stimulation of A_3_AR induced the strongest changes, with substantial increase of most pro-angiogenic cytokines analyzed (P<0.05). The most distinct was upregulation of bFGF, an autocrine growth factor for melanoma [24]. Only VEGF was significantly downregulated (P<0.05) to the level present in WT tumors with comparable iMVD, indicative of the changes in tumor vasculature. Stimulation of either A_2A_AR or A_1_AR induced not only comparable downregulation of VEGF, but also of G-CSF. Furthermore, stimulation of A_2A_AR upregulated bFGF to the level present in WT tumors, significantly when compared to both background controls.

Therefore, all three analyzed adenosine receptors are involved in regulation of pro-angiogenic proteins expression, with A_3_AR having the most significant role. The heat maps of the unsupervised-hierarchical clustering of the complete dataset of eighty-one angiogenic factors expression profiles (in S1 Fig) show similar trends. To some surprise, VEGF was not up-regulated through analyzed adenosine receptors. Additionally, it was not VEGF but rather bFGF which correlated with the changes in tumor microvascular density in B16F10 melanoma.

### Specific activation of mainly A_3_ but also A_2A_ and A_1_ adenosine receptor up-regulates macrophage infiltration of B16F10 melanoma and modulates macrophage polarization

**Fig 4:**
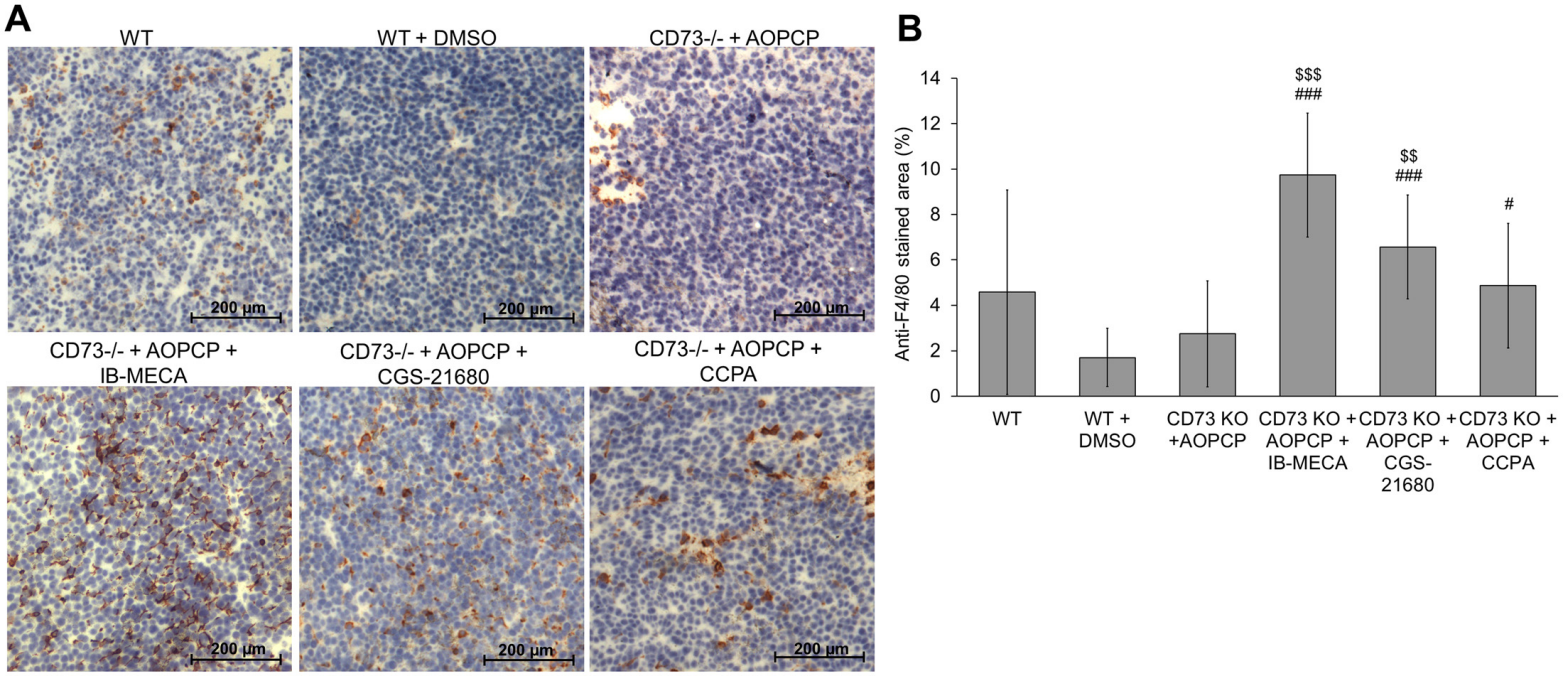
Stimulation of A_3_, A_2A_ or A_1_ adenosine receptors increases tumor infiltration by macrophages. (A) ICH staining of the monocyte/macrophage marker F4/80 in tumors isolated on the 14^th^ day of s.c. growth from WT or CD73^-/-^ mice. CD73^-/-^ mice were treated with AOPCP and, where indicated, with specific adenosine receptor agonist, IB-MECA (agonist for A_3_AR), CGS-2168 (A_2A_AR) or CCPA (A_1_AR). For separate control of agonist solvent, WT mice were treated with DMSO. Scale bar represents 200 μm. (B) Macrophage infiltration presented as a percentage of the surface stained with a marker from four to six fields per slide (3 mm^2^/field) from 3 tumors per a group. ^#^P<0.05, ^###^P<0.001 vs. WT + DMSO, ^$ $^P<0.01, ^$ $ $^P<0.001vs. CD73^-/-^ + AOPCP.

The role of adenosine signaling in macrophage infiltration was assessed with anti-F4/80 (a monocyte/macrophage marker) immunohistochemical staining in tumors isolated on the 14^th^ day from s.c. injection. It was quantified as the percentage of surface stained (4–6 x 3 mm^2^ fields per a slide) (Fig 4A and 4B).

We have already demonstrated that AOPCP significantly inhibits macrophage infiltration of B16F10 melanoma in CD73^-/-^ mice [4]. Comparable low macrophage presence in tumors from DMSO-treated WT mice (1.7% as compared to 2.7% in CD73-depleted mice) was also expected as DMSO is known to inhibit macrophage infiltration [18]. Due to an uneven distribution of macrophages in WT tumors (ab. 4.6% of surface stained), no significant differences in tumor infiltration compared to this control group were observed.

Compared to DMSO-treated WT mice and AOPCP-treated CD73^-/-^ mice, stimulation of A_3_AR in CD73-depleted mice significantly increased macrophage infiltration of the tumor (9.7%, P<0.001 for both) whereas A_2A_AR stimulation had slightly lesser effect (6.6%, P<0.001 and P<0.01, respectively). A_1_AR stimulation was the least efficient in increasing macrophage infiltration (4.8%, P<0.05 compared to DMSO-treated WT control). Macrophage infiltration positively correlated with microvessel density in agonist-treated tumors.

**Fig 5:**
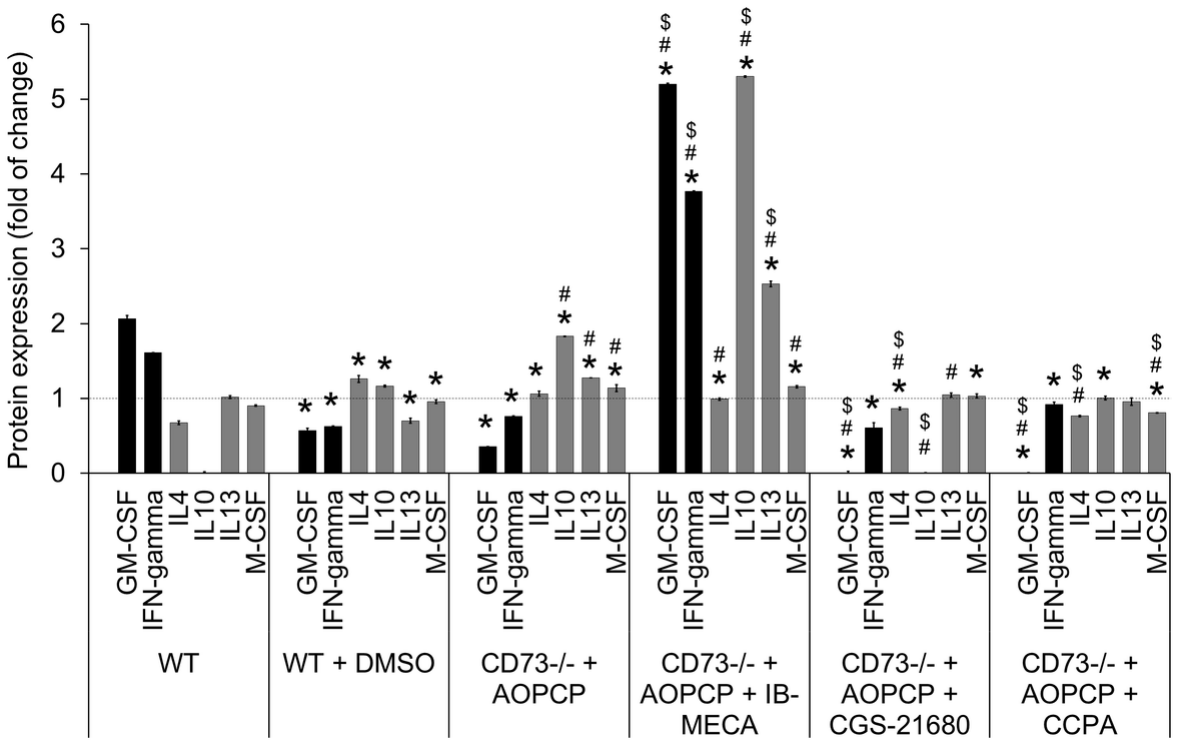
Stimulation of A_3_, A_2A_ or A_1_ adenosine receptors induces changes in cytokines involved in macrophage polarization. Cytokines were quantified in tumor lysates on the 14^th^ day of s.c. growth in WT or CD73^-/-^ mice. CD73^-/-^ mice were treated with AOPCP and, where indicated, with specific agonist: IB-MECA, CGS-2168 or CCPA. For separate control of agonist solvent, WT mice were treated with DMSO. Semi-quantitative antibody microarrays were used to obtain protein expression profile. Graph shows the expression of the selected cytokines involved in the polarization to the MI or MII phenotype (black and gray bars, respectively) presented as the fold changes between groups calculated from mean values for each protein separately assuming that the average expression of such protein in all three control groups is equal to 1. *P<0.05 vs. WT, ^#^P<0.05 vs. WT + DMSO, ^$^P<0.05 vs. CD73^-/-^ + AOPCP (other statistical differences are not shown).

To analyze the changes in factors influencing macrophage polarization in tumor microenvironment, a semi-quantitative analysis was performed with Mouse Cytokine Antibody Arrays (RayBiotech, Norcross, USA). Analyzed was the expression of GM-CSF (granulocyte-macrophage colony-stimulating factor) and IFN-gamma, main cytokines that can stimulate macrophage towards pro-inflammatory MI phenotype as well as interleukins -4, -10, -13 and M-CSF (macrophage colony-stimulating factor), that regulate polarization to pro-angiogenic MII phenotype [25, 26]. The fold changes in expression were calculated with 1 equal to the average expression of each protein in all three control groups (Fig 5).

Both DMSO treatment of WT mice and CD73 depletion led to a similar decrease in expression of pro-MI cytokines (P<0.05) and an increase in expression of pro-MII cytokines, correlating with an increased hypoxia in these tumors. Stimulation of A_3_AR led to a significant increase in pro-MI (GM-CSF, IFN-gamma) and in some pro-MII cytokines (IL-10, IL-13) (P<0.05 compared to all control groups). Both A_2A_AR and A_1_AR stimulation significantly (P<0.05, compared to all controls) downregulated the level of GM-CSF. Such stimulation also changed distribution of some pro-MII cytokines, in case of A_2A_AR mainly decreasing IL4 and IL10 expression and M-CSF expression when it was A_1_AR.

The above indicates an effective involvement of all three analyzed adenosine receptors, especially A_3_, in regulation of macrophage infiltration and macrophage polarization towards MII pro-angiogenic phenotype to counteract a hypoxia. It also reveals AR crucial role in regulation of macrophage pro-inflammatory MI phenotype, with A_3_AR transducing an activatory signal, but A_2A_AR and A_1_AR exerting some inhibitory effects.

## Discussion

### Role of adenosine receptors A_1_, A_2A_ and A_3_ in B16F10 melanoma growth

Deficiency of ecto-5’-nucleotidase (CD73), a key enzyme in extracellular cascade generating interstitial adenosine, was shown to decrease the rate of melanoma growth [3, 5, 17]. The change correlated with down-regulation of ERK1/2, a downstream part of mitogen-activated protein kinase pathway [4]. We demonstrate here, that such down-regulation in CD73-depleted B16F10 melanoma could be completely reversed by specific activation of any high affinity adenosine receptor. It was expected, as all adenosine receptors have been associated mainly with ERK1/2 activation [27, 28]. However, this reversal was observed at the late stage of tumor growth whereas at the earlier one any AR stimulation led to a significant decrease in tumor growth. This could be explained by a biphasic effects of AR activation observed *in vitro* in tumor cell lines [9, 10].

Especially activation of A_3_AR was shown to inhibit proliferation, e.g., in human A375 melanoma cells [10, 29]. Tumor cell-cycle arrest was indicated to be a result of a decreased intracellular cAMP and down-regulated PKB/Akt pathway [28]. This could explain changes in B16F10 melanoma growth induced by activation of A_3_AR or A_1_AR, but not by activation of A_2A_AR as it is connected with cAMP increase. However, cAMP can induce diverse effects in melanoma including inhibition of proliferation [30, 31]. Furthermore, A_2A_AR was also found to activate MAP kinases via cAMP-independent pathway with quick desensitization upon stimulation [32]. It is also possible that in CD73-depleted mice model, the activation of a single AR could be insufficient to stimulate cell proliferation at the early stage of tumor growth, with much less abundant tumor-derived growth factors. This suggests a complex role of extracellular adenosine in melanoma growth with possible stage-of-growth dependent temporal regulation.

Influence of dimethyl sulfoxide (DMSO) used as a solvent for agonists was also taken under consideration as it possesses various biological activities especially when applied intraperitoneally [18]. DMSO is able to activate ERK1/2 *in vitro* [33, 34]. However, its relatively low dose of 0.022 g/kg (0.02 ml/kg), caused no significant effect on tumor growth when injected into WT animals and also did not change ERK1/2 status compared to WT control. It proves that its direct influence is unlikely.

### A_1_, A_2A_ and A_3_ adenosine receptors in tumor neovascularization

Tumor vascularization was analyzed at the later stage of B16F10 melanoma growth in CD73-depleted mice, when activation of either A_1_, A_2A_ or A_3_ receptor lost its inhibitory effect on tumor growth rate. Increased tumor size correlates with elevated demand for oxygen and nutrients, and if not matched by angiogenic impulse, creates hypoxic microenvironment [35]. Accumulated extracellular adenosine is then an important hypoxia-counteracting metabolite generating a range of tissue responses including regulation of angiogenesis. Deficiency of ecto-5’-nucleotidase (CD73) was shown to slow vascularization of B16F10 melanoma in some reports [1, 17] but have no effect in others [5, 36]. However, the total depletion of CD73 resulted in a substantial decrease of intratumoral microvessel density (iMVD) [4]. Here we demonstrate, that A_3_AR activation completely reversed effect of CD73 depletion on tumor vascularization, resulting in significant decrease in expression of Bad, a hypoxia marker [20, 21]. Effect on iMVD was dependent on the type of receptor activated, as other receptors did not induce the full reversal of changes. Agonists were able to induce increase in tumor vascularization even in the presence of DMSO, a solvent with known anti-angiogenic activity including, e.g., decrease of VEGF and pro-angiogenic matrix metalloproteinase-2 expression (observed also in this report, S1 Fig) and inhibition of endothelial cell proliferation [22, 37, 38].

### A_1_, A_2A_ and A_3_ adenosine receptors in tumor angiogenesis

Direct correlation between level of CD73, intratumoral MVD and vascular endothelial growth factor (VEGF) was demonstrated by Allard and coworkers [1] in murine model of a breast cancer. It was then suggested that CD73 *via* its enzymatic activity enhances the expression of VEGF required for tumor angiogenic response. However, in B16F10 melanoma we observed that an increase in VEGF expression correlated mostly with increased hypoxia in CD73-depleted tumors having significantly reduced iMVD [4]. Hypoxia is able to increase expression of pro-angiogenic factors [8] including VEGF, even through a pathway independent of adenosine-regulated HIF-1 activation [39]. Here we confirm this observation demonstrating that iMVD increase induced through A_1_, A_2A_, or A_3_ receptors in CD73-depleted tumors did not correlate with upregulation of VEGF. Even when A_3_ receptor activation induced significant upregulation of other angiogenic factors, the level of VEGF was comparable to that in WT and below that in CD73-depleted tumors. However, the role of low affinity A_2B_AR as main regulator of VEGF expression as indicated by Ryzhov and coworkers [13] or the involvement of VEGF in vascularization at the early stage of B16F10 growth cannot be completely excluded.

Nevertheless, we have already [4] suggested that some effects of adenosine signaling on tumor vascularization in B16F10 melanoma may be mediated not only directly by influencing VEGF expression but by the regulation of VEGF/VEGF-R2 interactions and as such, the strength of angiogenic response. We observed, that in CD73-depleted B16F10 melanoma, the presence of VEGF-R2 on CD31-positive blood vessels was significantly decreased [4]. This decrease was reversed by stimulation of A_1_ and partially of A_2A_ and A_3_ receptors. This supports our suggestion, that extracellular adenosine takes part in up-regulation of VEGF-R2 presence on endothelium surface. There was, however, a lack of correlation between the amount of VEGF-R2 expressing blood vessels and its total expression (data in S1 Fig). When A_2A_ receptor was activated in CD73-depleted tumors, VEGF-R2 total expression was significantly decreased but activation of A_3_ receptor led to its strong increase. The role of A_2A_ receptor activation in reduction of VEGF-R2 expression at mRNA level was shown by Takagi and coworkers [40]. Taken together, it seems that VEGF-R2 protein expression could be both up- and down-regulated by adenosine signaling depending on the combination of adenosine receptors expressed, but VEGF-R2 internalization is down-regulated by all high affinity adenosine receptors. As a result, VEGF pro-angiogenic effect on endothelium via more effective VEGF/VEGF-R2 interactions could be augmented even when VEGF itself is not elevated. Alternatively, the mechanism could include VEGF-independent transactivation of VEGF-R2 directly by purinergic receptor as already indicated for P2Y receptors [41]. Any of those mechanisms needs to be confirmed, but our results point out activation of A_1_ receptor as the main starting point.

Adenosine can also stimulate proliferation of endothelial cells independently of VEGF, through other pro- and anti-angiogenic growth factors [11]. It was shown to induce release of pro-angiogenic interleukin-8 and bFGF from endothelial cells *via* activation of A_2B_ receptors [7, 14], and anti-angiogenic thrombospondin-1 via A_2A_ receptor [42]. We demonstrated the importance of all three high affinity receptors in regulation of this aspect of adenosine function. We also presented the prevalent role of A_3_AR with its much broader and pleiotropic influence on angiogenic factors expression in tumor development than already indicated. Additionally, we observed significant correlation between intratumoral microvessel density and expression of bFGF in agonists-stimulated tumors. Therefore, bFGF, a pro-angiogenic autocrine growth factor in melanoma [24], manifests here as the main candidate to mediate adenosine effect on tumor vascularization. bFGF could be also responsible for an increased proliferative potential of agonist-stimulated B16F10 tumors. It is also an important factor stimulating chemotaxis of macrophages [25].

### A_1_, A_2A_ and A_3_ receptors influence tumor infiltration by macrophages

Tumor associated macrophages (TAMs) with anti-inflammatory and pro-angiogenic MII phenotype accumulate in avascular and necrotic areas of solid tumors and promote tumor invasive potential and vascularization through secretion of growth factors, mainly VEGF [25, 43, 44]. In highly hypoxic B16F10 melanoma grown in CD73-deficient mice, we have observed downregulation of cytokines stimulating polarization to MI phenotype and hypoxia-driven upregulation of cytokines stimulating MII phenotype [4]. Above changes were also accompanied by significantly decreased macrophage infiltration. We attributed it to possible dysregulation of macrophage function due to deficiency in adenosine signaling. Yegutkin with coworkers [5] suggested that such deficiency could disturb macrophage activity and differentiation. Here, we demonstrated similar changes induced in WT mice grown tumors by DMSO, a known anti-inflammatory compound antagonistic to GM-CSF and able to decrease macrophage proliferation [45] and inhibit TAMs polarization to MII phenotype [46].

In our work, an increase in macrophage infiltration was evident upon stimulation of high affinity AR in CD73-deficient tumors, mainly A_3_ and A_2A_. Others found an activation of A_2A_AR [47] or A_3_AR [48] inhibitory to macrophage infiltration but in WT mice. Besides, these results were obtained for inflammatory and not neoplastic disease, where the balance between MI and MII subpopulations is disturbed, leading to the immune dysfunction and uncontrolled tumor progression [25, 49]. Lack of adenosine signaling could also led to an additional disturbance in macrophage polarization dysregulating macrophage response to a single adenosine receptor activation as observed in disproportionated response to A_3_AR activation.

Crucial roles of A_2B_ adenosine receptor in upregulation of MII macrophage activation and A_2A_ receptor in downregulation of MI phenotype were proposed [50, 51]. We observed, however, that in CD73-deficient tumors, activation of A_2A_AR have decreased expression of cytokines stimulatory to MI phenotype in tumor environment but it also changed expression of cytokines stimulatory to MII phenotype. Similar changes were also induced by activation of A_1_AR. Signaling through A_3_ receptor caused significant upregulation of cytokines stimulatory for both MI and MII macrophage polarization. Therefore, it is possible, that the regulation of macrophage polarization could involve a tight coordination of cross-interactions between various receptors.

## Conclusions

We can conclude that adenosine fulfils an important role in regulation of B16F10 melanoma vascularization with an outcome depending on the type of receptor activated. We suggest more complex role of this compound in regulation of tumor vascularization, besides observed *in vitro* direct mitogenic effects on vascular cells and ability to regulate the production of angiogenic factors. It includes a significant role in angiogenic activation of blood vessels executed through A_1_ receptor and significant role in tumor macrophage infiltration and macrophage polarization. It also indicates feasibility of recently proposed anti-CD73 cancer therapy [52] not only through its influence on immune system but also on tumor vascularization, growth and extravasation during metastasis formation. Further research in the role of adenosine receptors in tumor progression *in vivo*, especially in macrophage activation, should increase our understanding of possible biological factors limiting clinical application of CD73 inhibitors and insight into the role of adenosine signaling in tumor progression.

## Supporting Information

S1 Fig The dataset presenting fold changes in the expression profiles of angiogenesis-related proteins as heat maps. Heat maps of the unsupervised-hierarchical clustering of the data are separated into the expression profiles of pro-angiogenic and anti-angiogenic factors. Protein expression was analyzed in lysates from tumors at the 14^th^ day of growth in WT or CD73^-/-^ mice. CD73^-/-^ mice were treated with AOPCP and, where indicated, with specific adenosine receptor agonist, IB-MECA (agonist for A_3_AR), CGS-2168 (A_2A_AR) or CCPA (A_1_AR). For separate control of agonist solvent, WT mice were treated with DMSO. Samples were pooled from three tumors per group from three separate experiments. Semi-quantitative RayBio Mouse Cytokine Antibody Arrays 3 and 4 (G-series) for 62 and 34 cytokines were used to obtain protein expression profile, but only 81 factors that showed significant differences (p<0.05, Student’s t-test) in the expression between groups were analyzed. Each profile represents the fold changes between the means of the densitometric units from the antibody array where each protein was doubly spotted. Row normalization was used. Red denotes up-regulation and blue down-regulation. Experimental groups are also reordered based on their correlations according to the dendrogram on the top. Clusters go from root at left to leaf node for each cytokine. The branch shows the similarity, and the more similar, the shorter the branch.
(TIF)
