# Supplementary figures and images for: Specific Activation of A3, A2A and A1 Adenosine Receptors in CD73-Knockout Mice Affects B16F10 Melanoma Growth, Neovascularization, Angiogenesis and Macrophage Infiltration
Source: PLoS One. 2016 Mar 10;11(3):e0151420. doi: 10.1371/journal.pone.0151420 (PMC4786137; doi:10.1371/journal.pone.0151420)

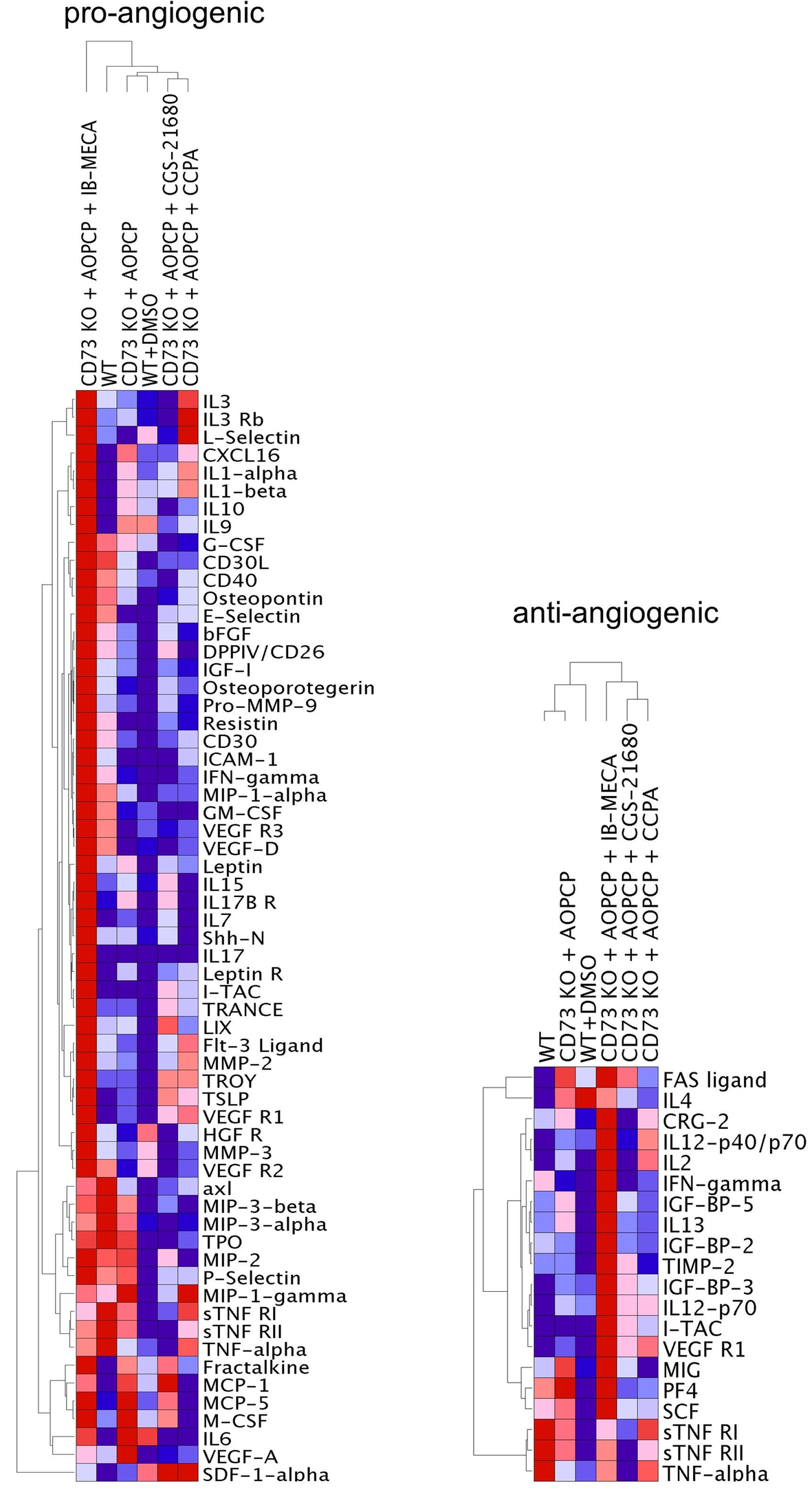

Supplement: S1 Fig — Heat maps of the unsupervised-hierarchical clustering of the data are separated into the expression profiles of pro-angiogenic and anti-angiogenic factors. Protein expression was analyzed in lysates from tumors at the 14th day of growth in WT or CD73-/- mice. CD73-/- mice were treated with AOPCP and, where indicated, with specific adenosine receptor agonist, IB-MECA (agonist for A3AR), CGS-2168 (A2AAR) or CCPA (A1AR). For separate control of agonist solvent, WT mice were treated with DMSO. Samples were pooled from three tumors per group from three separate experiments. Semi-quantitative RayBio Mouse Cytokine Antibody Arrays 3 and 4 (G-series) for 62 and 34 cytokines were used to obtain protein expression profile, but only 81 factors that showed significant differences (p<0.05, Student’s t-test) in the expression between groups were analyzed. Each profile represents the fold changes between the means of the densitometric units from the antibody array where each protein was doubly spotted. Row normalization was used. Red denotes up-regulation and blue down-regulation. Experimental groups are also reordered based on their correlations according to the dendrogram on the top. Clusters go from root at left to leaf node for each cytokine. The branch shows the similarity, and the more similar, the shorter the branch. (TIF) [file pone.0151420.s001.tif]
